# A POA-QPSO Hybrid Algorithm for Multi-Objective Optimization of Dual-Layer Walker Constellations

**DOI:** 10.3390/s26041391

**Published:** 2026-02-23

**Authors:** Yinuo Wang, Hongyuan Ye, Tianwen Du, Xuchu Mao

**Affiliations:** 1 School of Sensing Science and Engineering, Shanghai Jiao Tong University, Shanghai 200240, Chinayhyuan709711@sjtu.edu.cn (H.Y.); 2 School of Information Science and Engineering, Nanjing Audit University Jinshen College, Nanjing 210023, China

**Keywords:** low earth orbit (LEO) constellation, multi-objective optimization, pelican optimization algorithm (POA), quantum particle swarm optimization (QPSO), navigation augmentation

## Abstract

**Highlights:**

**What are the main findings?**

- The proposed hybrid POA-QPSO algorithm, utilizing a probability-driven dual-phase search mechanism, significantly outperforms MOPSO and MOPOA in convergence accuracy and solution diversity across ZDT benchmark functions.
- The optimized dual-layer Walker constellation (144 satellites at 800 km/50° and 56 satellites at 1426 km/82°) achieves 92.7% global coverage and an average PDOP of 1.78, superior to traditional single-layer configurations.

**What are the implications of the main findings?**

- The integration of diversity-triggered quantum behavior effectively overcomes premature convergence in high-dimensional discrete search spaces, providing a robust framework for solving complex aerospace engineering optimization problems.
- The study presents a cost-effective, high-precision architectural blueprint for next-generation LEO navigation augmentation systems, ensuring enhanced service availability in challenging environments such as urban canyons and polar regions.

**Abstract:**

The rapid development of low earth orbit (LEO) satellite constellations for navigation augmentation represents significant challenges in optimizing coverage performance while minimizing system complexity. A hybrid optimization algorithm based on pelican optimization algorithm and quantum particle swarm optimization (POA-QPSO) is proposed in this paper for multi-objective optimization design of dual-layer Walker constellations. The algorithm integrates the global search capability of the POA and the local exploitation ability of QPSO, effectively balancing exploration and exploitation through a probability-driven dual-phase search mechanism, a three-tier adaptive parameter adjustment strategy, and a pareto frontier maintenance mechanism. Probability factor and quantum tunneling facilitate low-cost deep search in complex non-convex environments. Experiments demonstrate that the algorithm outperforms MOPOA and MOPSO on ZDT test functions, with an 18.5% improvement in IGD metrics. In LEO constellation optimization, the designed dual-layer configuration (800 km/144 satellites in the first layer and 1426 km/56 satellites in the second layer) achieves a 92.7% global coverage, with an average PDOP of 1.78 and 5.8 visible satellites in polar regions. Furthermore, comparative benchmark tests show that the proposed solution outperforms most mainstream algorithms and performs better than traditional medium Earth orbit satellite systems in mid-to-high latitude regions. This research provides an efficient solution for LEO navigation augmentation system design.

## 1. Introduction

With the rapid advancement of information technology and the increasing demand for global connectivity, low earth orbit (LEO) satellite constellation systems have emerged as critical infrastructure for next-generation space–terrestrial information networks. The recent deployment of commercial LEO constellation projects, including SpaceX Starlink, Amazon Kuiper, and OneWeb, reflects a paradigm shift toward satellite-based Internet services [1]. Simultaneously, the transition toward 6G space–air–ground integrated network architectures requires enhanced technical capabilities from LEO satellite systems, particularly in achieving comprehensive global coverage, reducing communication latency, and providing robust navigation augmentation [2].

Although traditional global navigation satellite systems (GNSS) deliver fundamental positioning, navigation, and timing services via medium and high Earth orbit satellites, they suffer notable performance degradation in challenging environments. Signal obstruction and accuracy deterioration remain persistent issues in urban canyons, densely forested areas, and polar regions [3,4]. LEO-based navigation enhancement constellations offer a promising solution, as their proximity to ground users results in stronger signal strength and faster geometric configuration changes. Coupled with shorter ionospheric propagation paths, these features significantly improve GNSS availability, continuity, and positioning accuracy [5].

The Walker constellation configuration has gained widespread adoption in satellite system design due to its favorable global coverage characteristics and operational efficiency [6]. Its inherent geometric symmetry offers significant engineering advantages, including simplified orbit maintenance protocols and a reduction in propellant consumption by 30–40%, thereby extending satellite operational lifetimes. Additionally, Walker constellations achieve optimal satellite utilization efficiency, supporting continuous 1-fold to 4-fold global coverage, particularly in low-latitude and mid-latitude regions [7]. However, contemporary space mission requirements increasingly demand performance optimization across multiple dimensions, which single-layer Walker constellations cannot fully satisfy. Multi-layer constellation architectures, employing strategic satellite deployment across varying orbital altitudes, provide improved trade-offs among coverage performance, system capacity, and cost effectiveness, thus attracting substantial research interest. Specifically, dual-layer Walker constellations enhance regional service quality while maintaining global coverage continuity through optimized satellite distribution at different orbital altitudes.

Nonetheless, recent studies have revealed inherent theoretical limitations in Walker constellation optimization [8]. The expanded parameter space significantly increases computational complexity, while conventional analytical methods exhibit restricted capability in identifying global optima within multi-objective optimization frameworks. Constellation optimization design represents a complex multi-objective, multi-constraint problem involving numerous interdependent decision variables, such as orbital parameters, satellite quantity, ground coverage requirements, and system cost considerations. These challenges have driven the development of metaheuristic-based optimization approaches. Traditional single-objective optimization methods often fail to adequately address the inherent trade-offs among conflicting performance metrics. In contrast, multi-objective optimization algorithms generate Pareto-optimal solution sets, facilitating informed and balanced system design decisions [9].

In recent years, multi-objective optimization has become the dominant paradigm for LEO constellation design, as satellite systems are required to simultaneously balance conflicting performance metrics such as coverage continuity, positioning accuracy, satellite quantity, orbital altitude, and system cost [10]. Evolutionary algorithms including the non-dominated sorting genetic algorithm (NSGA-II), NSGA-III, and the multi-objective evolutionary algorithm based on decomposition (MOEA/D) have been widely applied to LEO navigation and augmentation constellation optimization, demonstrating their effectiveness in generating Pareto-optimal solution sets under competing objectives [7]. Moreover, dynamic multi-objective differential evolution algorithms have been introduced for two-layer and multi-layer Walker constellation configurations, yielding improved convergence behavior and coverage performance compared with classical genetic algorithms [11].

Recent advances in biomimetic intelligence have introduced innovative optimization paradigms. The pelican optimization algorithm (POA) mimics the collective foraging behavior of pelican colonies, exhibiting strong global search capabilities and rapid convergence characteristics. Likewise, the quantum-behaved particle swarm optimization (QPSO) algorithm integrates quantum mechanical wave function concepts to overcome local optima entrapment common in classical particle swarm methods, demonstrating enhanced performance in high-dimensional optimization problems [12]. Despite these advances, existing multi-objective optimization methods still face significant challenges when applied to large-scale, multi-layer Walker constellations. First, the rapid increase in decision-variable dimensionality substantially elevates computational cost and often leads to premature convergence or loss of population diversity in conventional evolutionary algorithms [10]. Second, many optimization frameworks assume relatively simple objective formulations and continuous feasible regions, whereas practical constellation design problems are subject to discrete constraints on satellite numbers, orbital planes, and altitude bands, resulting in fragmented and non-convex decision spaces [13]. Finally, most existing studies rely on a single optimization strategy, which limits their ability to simultaneously achieve robust global exploration and precise local exploitation in highly constrained multi-objective scenarios [14].

These limitations indicate a clear need for hybrid optimization frameworks that integrate complementary search mechanisms. By combining algorithms with distinct exploration and exploitation characteristics, hybrid approaches can improve convergence efficiency, enhance Pareto front diversity, and more effectively address the complex coupling relationships inherent in dual-layer Walker constellation design problems.

In this paper, we propose a hybrid POA-QPSO framework for the multi-objective design of dual-layer Walker constellations. The methodology synergistically leverages the global exploration capabilities of POA alongside the local exploitation strengths of QPSO, offering an efficient and precise optimization strategy for LEO navigation-enhanced constellation design. By systematically integrating these complementary algorithms, the research addresses the computational challenges inherent in multi-layer constellation optimization while achieving enhanced performance across multiple design objectives.

## 2. Walker Constellation Design and Multi-Objective Optimization Algorithm

### 2.1. Walker Constellation Configuration and Characteristics

The Walker constellation represents a classical symmetrical configuration paradigm in satellite system design. It achieves uniform spatial distribution of satellites through systematic regularization of orbital parameters, thereby ensuring stable and consistent coverage performance. This architectural approach emphasizes precise control of orbital elements, particularly the systematic variations in the right ascension of the ascending node (RAAN) and the argument of perigee (AOP), to establish optimal spatial distribution patterns.

**Figure 1:**
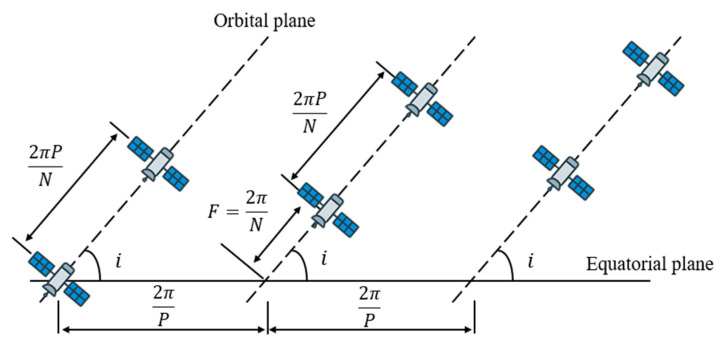
The orbital planes and satellite distribution of a Walker constellation.

Contemporary navigation satellite systems extensively employ Walker-based configurations. The global positioning system (GPS) has evolved through iterative optimization while preserving its fundamental Walker architecture. The European Galileo system is built on a standard Walker Delta 24/3/1 configuration, whereas the medium earth orbit segment of China’s BeiDou-3 constellation is founded on a Walker 24/3 pattern, a configuration shared by both GPS and Galileo. Similarly, the Iridium communication system employs an 86.4°: 66/6/2 Walker-Star configuration, specifically optimized for global voice communications, including coverage of polar regions. Mathematically, the Walker constellation is defined by the parameter set (T, P, F), where T denotes the total number of satellites, P represents the number of orbital planes, and F indicates the relative phase factor. Systematic optimization of these parameters allows flexible adjustment of regional satellite distribution while maintaining overall geometric symmetry. This parametric flexibility enables Walker constellations to achieve optimal coverage with a minimal number of satellites, establishing the architecture as a preferred choice for navigation and communication satellite system design. For a Walker constellation, the equatorial plane is used as the reference plane. A schematic diagram of the orbital planes and satellite distribution is provided in Figure 1, where N denotes total number of satellites in the constellation, i represents orbital inclination, the angle between the orbital plane and the equatorial plane.

In dual-layer constellation architectures, satellites are strategically deployed across distinct orbital altitudes to achieve complementary operational capabilities. The lower orbital layer, situated at reduced altitudes, delivers higher data transmission rates and minimal communication latency, making it particularly suitable for time-sensitive communication and navigation applications. In contrast, the higher orbital layer provides a broader coverage footprint and enhanced system redundancy. The synergistic interaction between these layers ensures an optimal balance among coverage performance, signal quality, and overall system reliability.

### 2.2. General Optimization Algorithms and Their Characteristics

Satellite constellation design and optimization research extensively employ evolutionary computing methods and swarm intelligence algorithms. Differential evolution (DE), proposed by Storn and Price in 1997, drives population evolution through differential mutation operations [15]. This method demonstrates robust performance in continuous optimization problems but remains sensitive to parameter configuration and often requires additional constraint-handling mechanisms, thereby increasing computational complexity.

Particle swarm optimization (PSO), proposed by Kennedy and Eberhart in 1995, is inspired by the behavioral modeling of avian foraging patterns [16]. PSO exhibits favorable convergence properties in continuous optimization tasks and has been successfully applied to Earth observation constellation orbit design. However, the algorithm is prone to premature convergence in high-dimensional problem spaces and demonstrates limited effectiveness in managing discrete variables.

In recent years, advances in optimization theory have led to the development of various novel metaheuristic algorithms, among which the POA and QPSO have attracted increasing attention due to their distinctive advantages in tackling complex satellite constellation optimization problems.

### 2.3. Pelican Optimization Algorithm (POA)

The POA achieves global optimization by biomimetically modeling pelican foraging behaviors in marine environments. The algorithm is characterized by minimal parameter requirements, structural simplicity, and rapid convergence. Its core principle simulates two distinct foraging strategies: surface exploration, corresponding to global search phases, and underwater predation, representing local exploitation phases. Through the synergistic coordination of these strategies, an effective balance between exploration and exploitation is achieved by POA. Its superior performance over traditional optimization methods, including GA, PSO, and DE, is consistently demonstrated across multiple benchmark test functions [11]. Nevertheless, given its relatively recent introduction, both theoretical analysis and practical applications of POA remain at an early stage.

#### 2.3.1. Local Search Mechanism (Pelican Foraging)

The exploration phase of POA employs a local search mechanism that simulates pelican flight trajectories during foraging activities. Within the dual-layer Walker constellation optimization framework, this mechanism efficiently explores diverse regions of the orbital parameter space, helping to prevent premature convergence to local optima.

The mathematical formulation of the local search mechanism is expressed as:

(1) $$x_{i}^{t+1}=\;x_{i}^{t}+\;r_{1}\times \;\left(x_{best}^{t}-\;x_{i}^{t}\right)\times cos\left(2\pi r_{2}\right)+\;r_{3}\times \;\left(x_{rand}^{t}-\;x_{i}^{t}\right)\times sin\left(2\pi r_{3}\right),$$

where $x_{i}^{t}$ denotes the position vector of the i-th pelican individual at generation t, $x_{best}^{t}$ represents the current optimal position, $x_{rand}^{t}$ indicates a randomly selected individual position, and $r_{1},r_{2},r_{3}$ are uniformly distributed random numbers in [0,1].

In dual-layer Walker constellation optimization, each dimension of the local search mechanism corresponds to specific orbital parameters. To maintain physical feasibility, the algorithm incorporates effective constraint-handling strategies:

(2) $$x_{i}^{t+1}=\;bound_{check}\left(x_{i}^{t}+\;spiral_{offset}\right),$$

where the $bound_{check}$ function enforces physical constraints including orbital altitude ranges and inclination limits, and$\;spiral_{offset}$ is the local search displacement generated by the spiral exploitation mechanism.

#### 2.3.2. Swarm Hunting Strategy

A swarm hunting strategy, which models the collaborative fishing behaviors of pelicans, is employed during the exploitation phase of the POA. This approach enables detailed exploration of promising regions through multi-individual cooperation, thereby improving solution quality.

The position update mechanism for swarm hunting is formulated as:

(3) $$x_{i}^{t+1}=\;x_{i}^{t}+\;\alpha \;\times \;\left(x_{center}^{t}-\;x_{i}^{t}\right)+\;\beta \;\times \;\left(x_{leader}^{t}-\;x_{i}^{t}\right),$$

where $x_{center}^{t}$ represents the current population centroid, $x_{leader}^{t}$ denotes the leader individual position, and α, β are adaptive weighting coefficients.

The centroid calculation incorporates fitness-based weighting:

(4) $$x_{center}^{t}=\frac{\Sigma \left(w_{i}\times \;x_{i}^{t}\right)}{\Sigma w_{i}},$$

where $w_{i}=\frac{1}{1+f_{i}}$, and $f_{i}$ represents the objective function value of individual i. This weighted centroid methodology allows individuals with superior fitness to exert greater influence on the centroid’s position, thereby guiding the search toward more promising regions.

This weighted-centroid mechanism is particularly suitable for multi-objective constellation design, as individuals with superior coverage and geometric performance exert stronger influence on the population’s exploitation direction.

#### 2.3.3. Exploration and Exploitation Balance

POA employs a dynamic probability Q to regulate the balance between exploration and exploitation:

(5) $$Q\;=\;\;\left(1\;-\frac{t}{T_{max}}\right)\times \;rand(),$$

where t denotes current iteration number, $T_{max}$ represents maximum iterations, and rand() generates uniform random numbers in [0,1].

When Q>0.5, the algorithm performs a local search (exploration); when Q≤0.5, swarm hunting (exploitation) is executed. This probabilistic switching mechanism aligns with the iterative refinement requirements of dual-layer constellation optimization, allowing extensive early-stage exploration of orbital architectures and focused late-stage refinement of promising Walker configurations.

### 2.4. Quantum Particle Swarm Optimization (QPSO)

QPSO, proposed by Sun et al. in 2005, integrates quantum mechanical principles into the traditional PSO framework [17]. The algorithm represents particle positions using quantum states and replaces classical Newtonian motion equations with Schrödinger equations for state updates. The need for velocity vectors is eliminated by this quantum-inspired approach, thereby reducing parametric complexity. Moreover, particles can move freely throughout the search space, significantly enhancing global search capabilities. QPSO has demonstrated superior performance in neural network training and structural optimization tasks. Compared to POA, QPSO reveals advantages in local search refinement and accelerated convergence, enabling rapid identification of high-quality solutions within promising regions. The complementary strengths of global exploration and local exploitation provide a solid theoretical basis for the development of hybrid optimization algorithms.

In the proposed framework, QPSO is not executed continuously; instead, it is selectively activated as an enhancement module when population diversity decreases.

#### 2.4.1. Quantum Behavior Modeling

Particle motion is modeled by QPSO using quantum mechanical principles. Unlike classical PSO, which follows deterministic trajectories, QPSO represents particle positions with a wave function that exhibits a probabilistic spatial distribution. The quantum state is described by the wave function ψ(x,t), where$\;{\left|\psi (x,t)\right|}^{2}\;$represents the probability density of finding a particle at position x. In one dimension, this can be modeled using a delta-potential well, which yields:

(6) $$\psi \left(x\right)=\;{\left(\frac{1}{L}\right)}^{\frac{1}{2}}\times \exp\left(-\frac{\left|x-C\right|}{L}\right),$$

where C denotes the potential well center position, and L represents the characteristic length parameter.

#### 2.4.2. Wave Function Collapse Mechanism

According to quantum measurement theory, measuring a particle’s position causes the wave function to collapse, thereby determining the particle’s location based on its probability distribution. This process is simulated by QPSO using Monte Carlo methods:

(7) $$x_{i}^{t+1}=\;C_{i}^{t}\pm \;L_{i}^{t}\times \ln\left(\frac{1}{u}\right),$$

where $u∼U\left(0,1\right)$ and the sign is chosen at random.

The potential-well center $C_{i}^{t}$ is computed as:

(8) $$C_{i}^{t}=\;\varphi \;\times \;p_{i}^{t}+\;\left(1-\varphi \right)\times \;g^{t},$$

where $p_{i}^{t}$ is the personal best of particle i, $g^{t}$ denotes global best position, and φ∼U[0,1].

#### 2.4.3. Mean Best Position Calculation

QPSO introduces the mean-best position (mbest), defined as the average of all personal best positions:

(9) $$mbest^{t}=\;\left(\frac{1}{N}\right)\times \;\Sigma \left(p_{i}^{t}\right),$$

where N denotes total particle count.

The characteristic length parameter $L_{i}^{t}$ computation incorporates mbest:

(10) $$L_{i}^{t}=\;2\;\times \;\alpha \;\times \;\left|mbest^{t}-\;x_{i}^{t}\right|,$$

where α denotes the contraction–expansion coefficient, which regulates the intensity of the quantum behavior.

Within the hybrid POA-QPSO framework, the QPSO component preserves population diversity through quantum-induced randomness, thereby mitigating the risk of premature convergence, enhances exploration across the search space by utilizing probabilistic position updates, and offers elite guidance via the mbest mechanism, harnessing collective intelligence to direct the search process.

The QPSO enhancement module is activated based on a population-diversity metric:

(11) $$diversity\;=\;\left(\frac{1}{N}\right)\times \;\Sigma \left|\left|x_{i}-\;x_{center}\right|\right|.$$

it is triggered when diversity<τ, where τ is a predefined threshold.

### 2.5. Adaptive Parameter Adjustment Strategy

Effective adaptive parameter adjustment relies on accurately identifying the convergence status. To achieve this, the algorithm employs a multi-metric fusion approach, enabling a comprehensive assessment of convergence.

The fitness improvement rate is calculated as:

(12) $$improvement_{rate}=\frac{\left(f_{best}^{t-w}-\;f_{best}^{t}\right)}{f_{best}^{t-w}},$$

where w denotes the sliding window size, f represents the fitness value of the objective function used in the optimization algorithm.

Population normalized diversity is measured through:

(13) $$normalized\text{\_}diversity\;=\;\sqrt{\left(\frac{1}{N}\right)\times \;\Sigma {\left|\left|x_{i}-\;x_{center}\right|\right|}^{2}}.$$

Search stagnation is indicated by:

(14) $$stagnation\;=\;\frac{generation\text{\_}without\text{\_}important}{\;total\text{\_}generations}.$$

The comprehensive convergence index integrates these metrics is explained as:

(15) $$convergence_{status}=w1\times \left(1-improvement_{rate}\right)+w2\times \;(1-(1-normalized\text{\_}diversity))+w3\times stagnation,$$

where w1, w2, w3 are weighting coefficients, satisfying w1+w2+w3=1.

Based on the determined convergence status, critical control parameters are adaptively tuned using linear-decay strategies.

Inertia weight adjustment q is:

(16) $$q\;=\;0.9\;-\;0.5\;\times \frac{gen}{maxGen}.$$

Cognitive coefficient adjustment c1:

(17) $$c1\;=\;2.5\;-\;1.5\;\times \frac{gen}{maxGen}.$$

Social coefficient adjustment c2:

(18) $$c2\;=\;0.5\;+\;1.5\;\times \frac{gen}{maxGen}\;.$$

This adaptive scheme prioritizes individual experience (larger c1) in the early iterations, while giving greater weight to collective intelligence (larger c2) in the later stages.

To preserve population diversity, a periodic mutation is implemented every 20 generations. This scheduled mutation strategy mitigates search instability caused by overly frequent mutations, while simultaneously providing extra exploratory momentum when the search shows signs of stagnation. Together, the adaptive adjustment and mutation mechanisms maintain a continuous balance between exploration and exploitation throughout the optimization process.

To balance global exploration and local exploitation, the proposed POA–QPSO hybrid algorithm adopts a probability-driven dual-phase search mechanism. At each iteration t, a Bernoulli random variable $I_{t}∼Bernoulli\left(P_{e}\right)$, is introduced to determine whether an exploratory POA phase is activated. When $I_{t}=1$, the population is first updated using POA-based exploration operators; otherwise, the algorithm directly proceeds to the QPSO exploitation phase. In this study, $P_{e}$ is set to a constant value of 0.5, enabling stochastic alternation between exploration and exploitation without imposing a fixed scheduling rule.

During the POA phase, particles perform large-scale exploratory moves inspired by pelican foraging behaviors, including attraction toward the current global best solution and randomized perturbations to promote population diversity. The magnitude of these perturbations is adaptively reduced as the iteration progresses, allowing the search process to gradually transition from coarse global exploration to finer local refinement.

Following the POA phase, all particles are updated using the QPSO mechanism as the exploitation phase of the hybrid framework. Each particle position is sampled around an attractor point defined by its personal best and the global best, according to a quantum-behaved position update rule.

Dual-layer Walker constellation optimization requires careful multi-objective trade-offs between coverage performance and system complexity. The POA-QPSO algorithm addresses this through Pareto dominance-based multi-objective processing, where Pareto dominance is formally defined, solution $x_{i}$ dominates $x_{j}$ is followed:

(19) $$\forall k\;\in \;\left\{1,2,\ldots ,m\right\}:\;f_{k}\left(x_{i}\right)\leq \;f_{k}\left(x_{j}\right),\;\exists l\;\in \;\left\{1,2,\ldots ,m\right\}:\;f_{l}\left(x_{i}\right)<\;f_{l}\left(x_{j}\right),$$

where m represents the objective function count.

Pareto front maintenance utilizes fast non-dominated sorting algorithms. This process ranks population individuals based on non-dominance relationships and dynamically updates Pareto-optimal solution sets in external archives throughout the evolution. When the archive exceeds its predefined capacity, crowding distance-based selection is applied to preserve both the representativeness and the distributional diversity of the solution set.

To maintain high-quality solutions throughout the evolutionary process, the algorithm employs elite retention:

(20) $$x_{i}^{t+1}=\;x_{i}^{t}+\;r_{1}\times \;\left(x_{best}^{t}-\;x_{i}^{t}\right)\times cos\left(2\pi r_{2}\right)+\;r_{3}\times \;\left(x_{rand}^{t}-\;x_{i}^{t}\right)\times sin\left(2\pi r_{3}\right),$$

where rank(x) represents the non-dominated sorting rank of individual x. Elite individuals are retained across generations and serve as guiding mechanisms in the POA and QPSO search processes.

**Figure 2:**
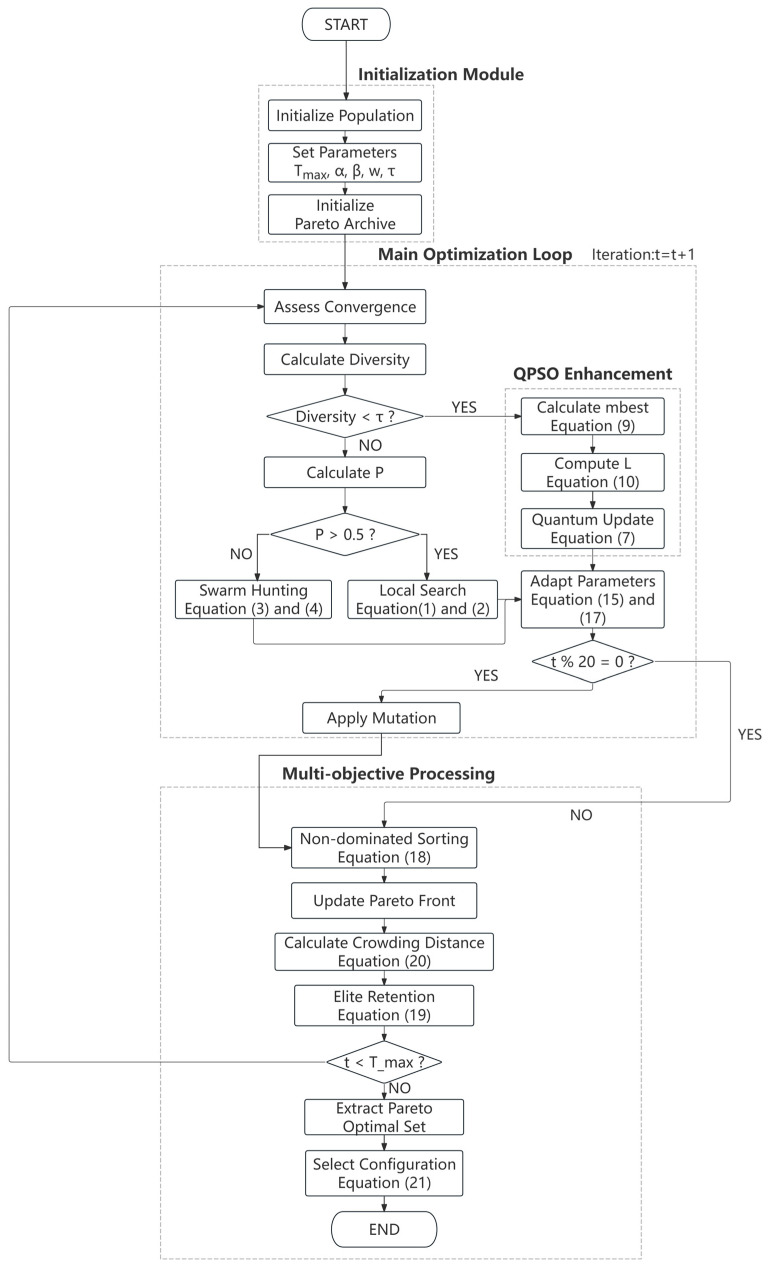
POA-QPSO algorithm flow chart.

To preserve diversity along the Pareto front, the crowding distance of each individual is calculated as:

(21) $$crowding\text{\_}distance_{i}=\frac{\;\Sigma \left(f_{k}^{i+1}-\;f_{k}^{i-1}\right)}{\;f_{k}^{max}-f_{k}^{min}},$$

where $f_{k}^{i+1}$ and $f_{k}^{i-1}$ are the objective-function values of adjacent individuals along the k-th objective. Individuals with larger crowding distances correspond to lower local density are thus preferentially selected during environmental selection, which helps preserve solution diversity. In practical applications, the final constellation configuration is chosen from the Pareto-optimal set using a weighted-sum decision strategy as:

(22) $$f_{combined}=\;\Sigma \left(w_{k}\times \;f_{k}\right),$$

where $w_{k}$ denotes the weight assigned to each objective. The algorithm flow chart is shown in Figure 2.

From a theoretical perspective, the proposed hybrid framework inherits the convergence properties of QPSO. QPSO can be interpreted as a contraction process in the probabilistic sense and converges asymptotically to the global optimum under appropriate parameter settings. By intermittently incorporating POA-based exploratory updates, the algorithm maintains sufficient population diversity, while the QPSO phase ensures stable convergence toward high-quality solutions. Consequently, the probability-driven dual-phase mechanism provides an effective and theoretically balance between exploration and exploitation.

## 3. Experiments on Standard Test Functions

### 3.1. Experimental Setup

To validate the effectiveness of our proposed POA-QPSO hybrid framework for multi-objective optimization, we utilize the ZDT benchmark function suite (ZDT1-ZDT6) introduced by Zitzler et al. [18] as experimental testbeds. These functions exhibit diverse characteristics, including convex Pareto fronts, non-convex and discontinuous frontiers, multimodal landscapes, and highly nonlinear structures, thereby enabling a comprehensive assessment of the algorithm’s performance in terms of convergence behavior and maintenance of solution diversity. Note that the ZDT5 function is excluded from this study as it represents a binary-coded discrete optimization problem.

The comparative evaluation includes two well-established benchmark algorithms that represent state-of-the-art approaches in multi-objective optimization: Non-dominated Sorting Multi-objective PSO (MOPSO), MOQPSO and MOPOA. Additionally, the widely used evolutionary algorithm NSGA-II is included to ensure a broad comparison. These methods are widely adopted in multi-objective optimization applications and serve as suitable baselines for performance assessment.

To ensure experimental reproducibility, the random seed is fixed at 42 for all trials. The experiments were conducted with a consistent population size of 500 individuals across all algorithms, and each run was terminated after 250 generations. The detailed parameter configurations for all algorithms are summarized in Table 1.

### 3.2. Performance Metrics

Algorithm performance is evaluated using three well-established metrics that provide a comprehensive assessment across multiple dimensions. Hypervolume (HV) quantifies the volume of objective space dominated by the non-dominated solution set, with larger values indicating superior solution quality. Inverted generational distance (IGD) measures the average distance between the obtained solution set and the true Pareto front, where smaller values reflect better convergence accuracy. Spacing assesses the uniformity of the distribution of non-dominated solutions along the Pareto front, with smaller values indicating a more balanced distribution. Statistical significance is ensured through 30 independent runs of each algorithm on each test function, with mean values and standard deviations reported for all metrics.

### 3.3. Experimental Results and Analysis

**Figure 3:**
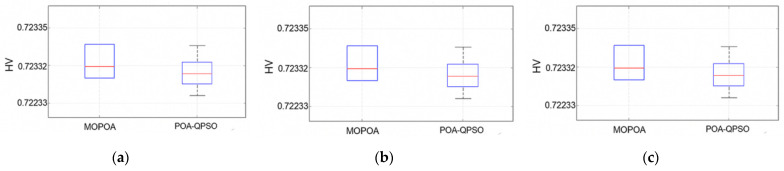
Distribution comparison of three performance metrics between MOPOA and POA-QPSO on the ZDT1. (**a**) Inverted Generational Distance (IGD), (**b**) Hypervolume (HV), and (**c**) Spacing. (Red: mean; Blue: standard deviation).

We compared the performance metrics of MOPOA and POA-QPSO on the ZDT1 problem, and the results are shown in Figure 3. The IGD value of the POA-QPSO algorithm is significantly lower, indicating that it is closer to the true Pareto front and has higher convergence accuracy. A similar trend was observed in the Spacing metric, with the spacing value of POA-QPSO being significantly lower, indicating that its solution is more evenly distributed on the Pareto front. In contrast, the HV metric did not show a statistically significant difference between the two algorithms, indicating that they are comparable in terms of hypervolume coverage. Similarly, we performed the same analysis on other ZDT datasets. The IGD results of benchmark functions are shown in Table 2, the spacing results of benchmark functions are shown in Table 3, and the HV results of benchmark functions are shown in Table 4.

On the ZDT1 benchmark, although the numerical improvement in HV values appears marginal, the statistical analysis confirms that POA-QPSO achieves a statistically significant advantage over the comparative baseline. Specifically, the Wilcoxon rank-sum test yields a *p*-value of $8.35\times 10^{-8}$, which is well below the 0.05 significance level. The extremely low *p*-value demonstrates that the incorporation of QPSO significantly enhances algorithmic robustness and exploitation precision. Consequently, POA-QPSO consistently locates a better-converged non-dominated solution set across independent runs, proving its stability even when the optimization space is easily accessible.”.

We notice that the MOPSO results for ZDT4 show much larger AVE and SD. ZDT4 contains numerous local minima that can trap swarm-based algorithms. Because MOPSO updates particle positions based on personal and global bests, its swarm is prone to being trapped in shallow basins on the multi-modal landscape, leading to a high average IGD. In contrast, QPSO employs a quantum-behavior diffusion model that allows particles to sample the search space more broadly, enabling escape from local optima and yielding superior convergence on ZDT4.

In terms of convergence accuracy, POA-QPSO exhibits distinct advantages. The algorithm achieves significantly lower average IGD values compared to MOPSO on both the convex ZDT1 and the challenging ZDT4 functions. Notably, approximately 80% of independent runs for POA-QPSO attained optimal IGD results, indicating robust approximation capabilities across both simple and complex fitness landscapes.

Analysis of the Spacing metric reveals that POA-QPSO produces highly uniform solution sets. Specifically on the ZDT4 function, POA-QPSO achieved Spacing values approximately 21% lower than those of MOPSO. This demonstrates the proposed algorithm’s ability to maintain a uniform distribution across discontinuous or multi-modal frontiers, avoiding the issue of excessive clustering in localized regions often observed in standard swarm algorithms.

The global coverage assessment across ZDT2, ZDT4, and ZDT6 further confirms the superiority of the hybrid framework. POA-QPSO consistently yielded high HV values. Most notably, on the non-convex ZDT2 function, POA-QPSO achieved an average HV value approximately 7% higher than MOPSO, substantially outperforming all benchmark algorithms. This result highlights the algorithm’s enhanced capability to provide comprehensive coverage of the Pareto front in complex, nonlinear multi-objective optimization problems.

The systematic evaluation across the ZDT test suite conclusively validates the efficacy of the POA-QPSO framework. By consistently surpassing baseline methods (MOPSO and MOPOA) in HV, IGD, and Spacing metrics across the majority of test cases, POA-QPSO is established as a robust and effective multi-objective optimization approach with considerable potential for solving complex engineering problems.

## 4. LEO Navigation Enhancement Constellation Simulation Experiments

### 4.1. Constellation Design Parameter Specifications

The design of LEO navigation enhancement constellations requires thorough consideration of orbital mechanics constraints, technical feasibility, and economic factors. We establish parameter ranges that reflect practical engineering limitations while allowing effective exploration during the optimization process. We summarize the key parameter as shown in Table 5.

These variables define the structure of the Walker constellation and its hybrids. The decision variable set for optimization, denoted as Params, is expressed as

(23) $${Params=[alt}_{w},\;{inc}_{w},N_{w},\;{planes}_{w},F_{w},{alt}_{p},\;{inc}_{p},N_{p},\;{planes}_{p},F_{p}],$$

where w represents the Walker configuration, and p represents the polar configuration.

As shown in Table 6, a layered collaborative architecture is implemented by this parameter design: the first layer is assigned the prioritization of signal strength and rapid geometric changes, while the second layer is entrusted with the assurance of coverage stability and global reach. This complementary structure enables the dual optimization of coverage performance and navigation accuracy within constrained resources.

To ensure a physically meaningful search space, the parameters in Table 6 are grounded in engineering constraints, including the 400 km IADC/FCC de-orbit limit and ESA debris avoidance (800–1000 km). Layer 1 targets the ±53° population belt using 50–70° inclinations, requiring 10–20 planes to ensure dense mid-latitude coverage at lower altitudes. In contrast, Layer 2 utilizes near-polar inclinations (80–90°) and higher altitudes (1000–1500 km); this geometry significantly expands the field of view, enabling seamless global coverage with only 3–6 planes, analogous to the Iridium architecture.

### 4.2. Optimization Algorithm Configuration

In the constellation optimization experiments, we utilize the POA-QPSO hybrid optimization algorithm, with carefully calibrated parameters designed to maintain an effective balance between exploration and exploitation throughout the optimization process.

In the POA component, a population size of 100 individuals is employed to ensure comprehensive coverage of the search space. The local search parameters $r_{1}$, $r_{2}$ and $r_{3}$ are stochastically assigned within the range [0,1], directing individual search trajectories across the solution space. Swarm hunting weights are dynamically adjusted, with α decreasing and β increasing proportionally to the iteration count, thereby promoting broad exploration during the early phases and focused exploitation in later stages. Additionally, the exploration probability threshold incorporates stochastic variations correlated with iteration progress, introducing controlled perturbations that enhance the robustness of the search process.

In the QPSO component, linearly decreasing contraction–expansion coefficients are employed to enable gradual convergence toward optimal solutions. A quantum behavior activation threshold of 0.1 initiates quantum mechanisms when population diversity drops below critical levels, thereby preventing premature convergence. The mbest position update integrates a random weighting factor, φ, which strengthens the algorithm’s ability to escape local optima.

Common parameters across both components include a maximum of 600 iterations, an exponentially decaying mutation probability to minimize perturbations during later search stages, a Pareto archive capacity of 200 for storing non-dominated solutions, and a 20-generation convergence assessment window to monitor the dynamic stability of the solution set. In the computation of the comprehensive convergence index, the weighting coefficients are set to w1=0.3, w2=0.4, and w3=0.3.

### 4.3. Performance Evaluation Framework

In this study, we establish a comprehensive evaluation framework that encompasses coverage performance, navigation accuracy, and system cost, enabling a multi-faceted assessment of constellation solutions.

Coverage performance is evaluated using four key metrics: global average coverage, China regional coverage, polar coverage, and coverage continuity. Coverage metrics are calculated as the ratio of the covered area to the total area, while coverage continuity is measured by the average continuous visibility duration. Together, these metrics provide a holistic characterization of constellation service capabilities across diverse spatial regions.

Navigation accuracy assessment focuses on position dilution of precision (PDOP), geometric dilution of precision (GDOP), visible satellite count, and signal strength. PDOP and GDOP are standard metrics that quantify the influence of satellite geometry on positioning errors in navigation systems. The visible satellite count provides a direct measure of the number of satellites accessible to the user, while signal strength reflects the quality of the received signals.

System cost evaluation encompasses three dimensions: total satellite count, launch cost, and operational complexity. The satellite count directly reflects the overall system scale, while launch cost is influenced by both the number of satellites and their orbital parameters. Operational complexity depends on the configuration of orbital planes and maintenance strategies, indirectly indicating the long-term sustainability of system operations.

The integration of these three evaluation categories creates a comprehensive assessment framework that balances system performance with cost considerations.

Regarding the cost constraints, the optimization algorithm utilizes a continuous cost function to ensure a smooth fitness landscape for effective search convergence. However, we acknowledge that practical engineering costs are quantized, as launch vehicles (e.g., Falcon 9) incur fixed prices per mission regardless of the payload fill ratio. Consequently, while the algorithm searches in a continuous space, the final deployment number (N) should practically be adjusted to an integer multiple of the rocket’s fairing capacity to maximize launch efficiency.

For the constellation performance evaluation, the target function integrates four key indicators with the following assigned weights: PDOP (0.30), number of visible satellites (0.25), spatial uniformity (0.25), and average altitude (0.20). It is important to note that these specific weighting coefficients were not derived in this study, but rather referenced a recently validated study that used the analytic hierarchy process (AHP) to process a large number of GNSS datasets [19]. According to this analysis, PDOP was given the highest priority because it directly amplifies ranging errors, while visibility and uniformity were considered as secondary factors.

### 4.4. Experimental Results

To objectively assess the performance of POA-QPSO, we selected MOPSO as benchmark algorithms, as they represent two widely adopted multi-objective optimization paradigms in constellation optimization. These algorithms offer multi-dimensional performance references through their distinct optimization strategies.

MOPSO enhances the traditional PSO framework by integrating an external elite archive alongside crowding distance maintenance mechanisms, allowing for dynamic preservation and updating of non-dominated solution sets.

All algorithms are implemented under identical experimental settings, including a population size of 30 individuals, 600 function evaluations, consistent boundary constraint handling, and uniform initialization strategies. The termination criterion is defined by the maximum number of iterations. To ensure statistical reliability, each algorithm is executed independently 10 times.

Experimental results reveal progressive optimization trends. The optimal PDOP value decreases from approximately 1.155 to 1.135, while the number of visible satellites increases stepwise from 20.5 to around 22.8. The average fitness exhibits notable volatility, fluctuating between 3 and 21, with pronounced spikes observed during generations 18–20, which may indicate changes in population diversity near convergence or transitions between algorithmic phases. Meanwhile, the diversity metric shows a general upward trend with significant oscillations, rising from near zero to approximately 130.

**Figure 4:**
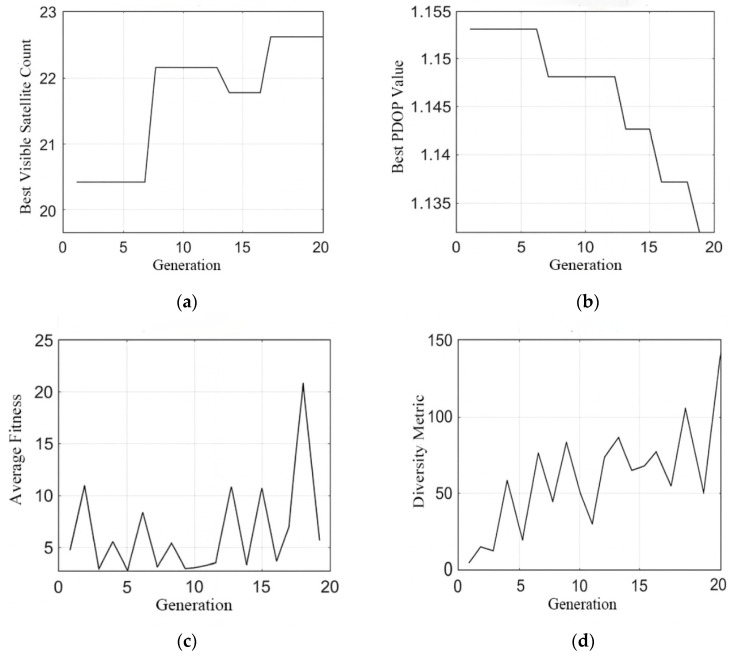
Convergence and evolutionary characteristics of the POA-QPSO process. (**a**) PDOP convergence history (**b**) Visible satellite count convergence history (**c**) Population mean fitness (**d**) Population diversity.

As illustrated in Figure 4, the POA-QPSO algorithm demonstrates a distinctive “step-wise” convergence profile combined with high-frequency fluctuations in population diversity (Figure 4d) and average fitness (Figure 4c). These characteristics indicate that the algorithm actively combats premature convergence throughout its entire lifecycle. The sustained high diversity in the late iterative stages (Generations 15–20) enables the algorithm to identify superior geometric configurations, resulting in a sharp reduction in the PDOP value to below 1.135, outperforming the initialization baseline significantly.

**Figure 5:**
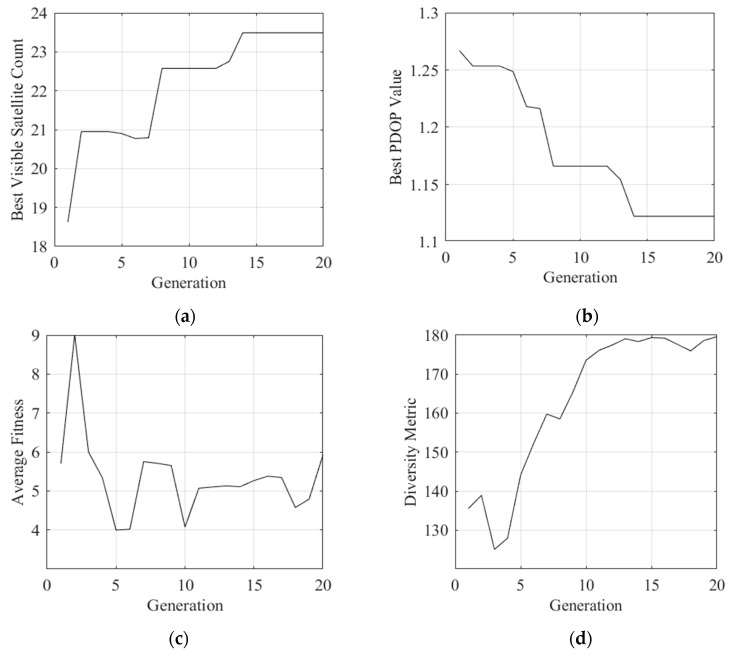
Convergence and evolutionary characteristics of the MOPSO optimization process. (**a**) PDOP convergence history (**b**) Visible satellite count convergence history (**c**) Population mean fitness (**d**) Population diversity.

In contrast, the MOPSO process depicted in Figure 5 exhibits a more traditional, monotonic convergence pattern. While the algorithm successfully reduces the PDOP value, the rapid stabilization of the average fitness metric (Figure 5c) and the saturation of the diversity metric (Figure 5d) after the 10th generation suggest a premature loss of exploratory capability. The smooth but plateauing curves indicate that MOPSO relies heavily on the inertia of historical best positions, lacking the intrinsic mechanism to escape strong local attractors in the high-dimensional search space. Consequently, POA-QPSO is empirically validated to possess superior global search robustness and efficiency in solving the combinatorial satellite selection problem.

POA-QPSO produces an optimal configuration that balances coverage and cost. Layer 1 is positioned at an altitude of 800 km with a 50.8° inclination, comprising 144 satellites, while Layer 2 is set at 1426 km with an 82° inclination, consisting of 56 satellites. This configuration achieves 92.7% global coverage and extends continuous coverage time over China to 11.8 h. Furthermore, integration with BeiDou-3 satellites enhances global coverage to 99.7%. PDOP coverage is defined as the percentage of time or service area wherein a receiver maintains simultaneous line-of-sight with at least four satellites and the PDOP value remains below a designated threshold.

As shown in Table 7, for China regional enhancement scenarios, the optimization results achieve 93.5% coverage within China’s territory, with the average PDOP reduced to 1.78, demonstrating substantial improvements in regional performance.

**Figure 6:**
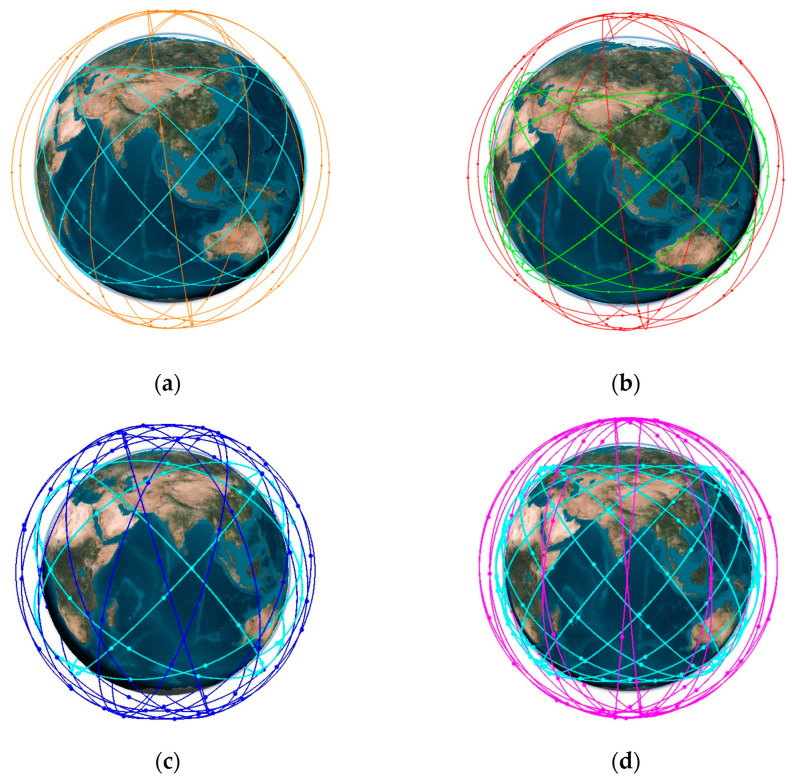
Spatial configuration of each optimization scheme. (**a**) POA-QPSO: Layer 1 (Cyan) and Layer 2 (Orange); (**b**) MOPSO: Layer 1 (Green) and Layer 2 (Red); (**c**) MOPOA: Layer 1 (Cyan) and Layer 2 (Blue); (**d**) NSGA-II: Layer 1 (Cyan) and Layer 2 (Magenta).

In polar communication scenarios, the dual-layer high-inclination design increases polar coverage to over 95% for both the Arctic and Antarctic regions, maintaining an average of 11.8 visible satellites to support polar research and communication requirements. The optimized space configuration is shown in Figure 6. The left and right sub-figures show the optimal orbit configurations obtained by different algorithms. Blue and green represent the first-level orbits, and orange and red represent the second-level orbits. The bottom sub-figure shows the constellation containing the BDS-3.

Pareto front analysis highlights the inherent trade-offs between coverage and cost, accuracy and complexity, and global versus regional performance, offering decision-makers a diverse set of configuration options.

**Figure 7:**
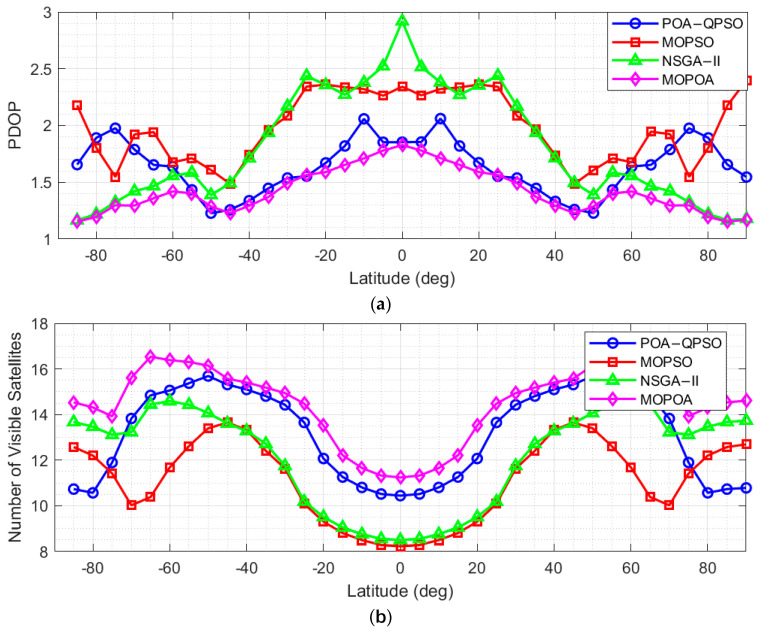
Performance Comparison among the NSGA-II, MOPSO, MOPOA, and the POA-QPSO Optimized LEO Constellation. (**a**) PDOP (**b**) Number of visible satellite.

Based on the optimization results, the best-performing LEO navigation constellation configuration obtained by each algorithm was selected, and its standalone navigation performance was evaluated through simulation. The simulations were conducted over a duration of one day with a time step of 60 s, covering the global region. The minimum elevation angle was set to 5°, and a 5° × 5° latitude–longitude grid was adopted. The number of visible satellites and the position dilution of precision (PDOP) were used as evaluation metrics, and the corresponding results are shown in Figure 6. In addition, the visible satellite count and PDOP of the POA-QPSO-optimized constellation were further compared with those of BDS-3 and GPS, with the results presented in Figure 7.

Figure 7 illustrates the latitude-dependent performance of dual-layer LEO Walker constellations optimized by POA-QPSO, MOPSO, NSGA-II, and MOPOA. The results indicate that the POA-QPSO solution (blue line) offers the most robust global coverage, maintaining a stable visible satellite count (fluctuating between 12 and 16) and consistently achieving the lowest PDOP values, particularly in mid- and high-latitude regions. In contrast, MOPSO (red line) and NSGA-II (green line) exhibit significant degradation characterized by low visibility and geometric instability near the equator and poles, respectively. Although MOPOA (purple line) yields coverage and geometric performance comparable to the proposed method, it necessitates a larger total number of satellites, resulting in greater total constellation mass and higher deployment costs. Consequently, POA-QPSO emerges as the most balanced solution, delivering optimal global performance with superior cost-effectiveness compared to the heavier MOPOA configuration.

**Figure 8:**
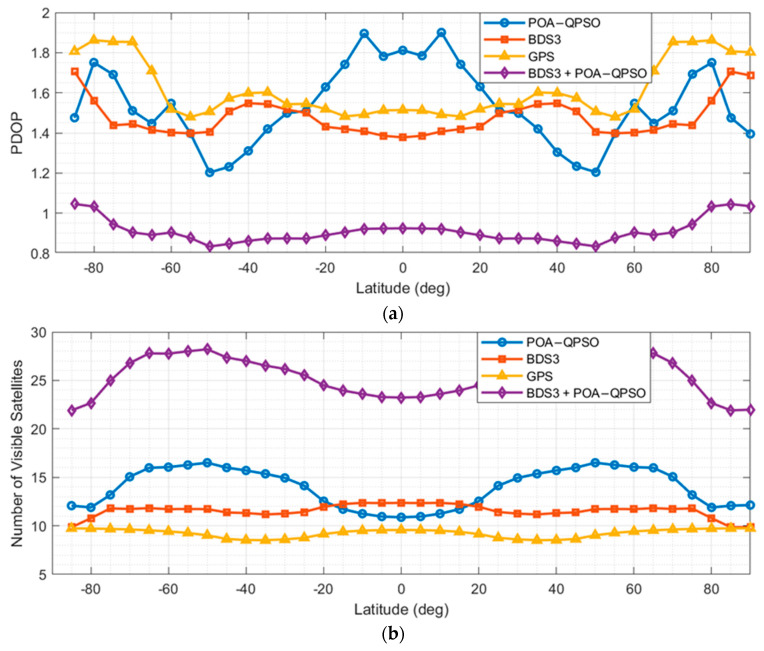
Performance Comparison among the BDS-3, GPS, and the POA-QPSO Optimized LEO Constellation. (**a**) PDOP (**b**) number of visible satellite.

Figure 8 benchmarks the latitude-dependent performance of the optimized POA-QPSO constellation against BDS-3, GPS, and a hybrid POA-QPSO/BDS-3 configuration. The hybrid architecture demonstrates global superiority, achieving the highest visible satellite counts and lowest PDOP values across all latitudes by leveraging the complementary geometry of LEO and high-orbit layers. Among standalone systems, the POA-QPSO constellation generally outperforms BDS-3 and GPS in coverage and geometric accuracy, particularly in mid-to-high latitudes. However, in low-latitude regions, BDS-3 maintains a geometric advantage (lower PDOP) due to its inclusion of high-altitude GEO and IGSO satellites, despite POA-QPSO offering comparable visibility. This indicates that while the proposed LEO constellation offers robust global performance, surpassing BDS-3’s geometric accuracy in equatorial zones would require further structural optimization, such as augmenting the number of inclined satellites.

## 5. Conclusions

In this paper, we propose a POA-QPSO hybrid optimization algorithm for the multi-objective optimization of dual-layer Walker constellations. For the first time, the algorithm integrates the POA with Quantum PSO, establishing a dual-layer search framework that dynamically balances global exploration and local exploitation through probability-driven mechanisms. This mechanism effectively achieves robust convergence in the space of the two-layer Walker constellation problem by dynamically adjusting the search strategy and using quantum behavior to escape only when population diversity is lost. This overcomes the defect of traditional static hybrid algorithms that are prone to getting trapped in local optima. A parameter-adaptive strategy based on convergence-state recognition incorporates the fitness improvement rate, population diversity, and search stagnation indicators to achieve precise monitoring of the algorithm’s operational states. The constructed Pareto-based multi-objective optimization framework combines fast non-dominated sorting, crowding distance, and elite retention strategies, ensuring both convergence of the solution set and uniform distribution along the Pareto front.

Experimental results demonstrate that POA-QPSO attains optimal performance in 5 out of 6 ZDT series test functions, with HV values improving by 7–12% on complex multimodal functions. The evaluation of the algorithm-designed dual-layer LEO constellation (144 satellites at 800 km; 56 at 1426 km) demonstrates a global coverage of 92.7%, and an average PDOP of 1.78, yielding a standard deviation of only 3.2% across 10 independent runs.

The navigation performance analysis further validated the robustness of the proposed framework. When benchmarking against MOPSO, NSGA-II, and MOPOA, the POA-QPSO solution exhibits stable visibility and PDOP distribution across all latitudes, avoiding the equatorial degradation seen in other algorithms while maintaining a lower total satellite mass than the comparable MOPOA configuration. Comparisons with established navigation systems indicate that the standalone LEO constellation surpasses GPS and BDS-3 in mid- and high-latitude coverage; however, a hybrid configuration combining POA-QPSO with BDS-3 delivers the optimal global performance, effectively mitigating geometric dilution in low-latitude regions and demonstrating the potential for LEO-augmented multi-layer navigation architectures.

Compared to traditional methods, POA-QPSO achieves satisfactory solutions within 600 iterations and reduces the total satellite count by approximately 15%. The Pareto solution set facilitates flexible trade-offs between performance and cost. Nevertheless, current research primarily addresses static configurations without fully accounting for dynamic factors. Future work should focus on extending dynamic reconfiguration capabilities, enhancing computational efficiency for large-scale constellations, and incorporating additional engineering constraints. Overall, this algorithm offers an efficient and reliable technical solution for LEO navigation augmentation systems.

## Figures and Tables

**Table 1:** Detailed parameter configurations.

Algorithm	Parameter	Value/Description
Common Settings	$\mathrm{Population}\;\mathrm{Size}\;(N_{p}$)	500
	$\mathrm{Repository}\;\mathrm{Size}\;(N_{r}$)	300
	$\mathrm{Max}\;\mathrm{Iterations}\;(G_{max}$)	250
	Independent Runs	30
	Levy Flight Parameter (β)	1.5
POA-QPSO	Stage Ratios	Phase 1 (POA): 0.4, Phase 2 (Hybrid): 0.6
	QPSO Contraction (α)	Linearly decreasing 1.0 to 0.5
	Elite/Opposition Prob.	0.3/0.3
MOQPSO	$\mathrm{Mutation}\;\mathrm{Distribution}\;(\eta_{m}$)	20
MOPSO	Inertia Weight (w)	Linearly decreasing from 0.9 to 0.4
	$\mathrm{Acceleration}\;(c_{1},c_{2}$)	1.5, 1.5
MOPOA	Random Integer (I)	$\mathrm{Round}\;\left(1+\mathrm{rand}\right)\in \{1,2\}$
	Exploration Coeff.	$0.2\times \left(1-\mathrm{gen}/G_{max}\right)$
NSGA-II	$\mathrm{Crossover}\;\mathrm{Prob}.\;(p_{c}$)	0.9
	$\mathrm{Mutation}\;\mathrm{Prob}.\;(p_{m}$)	1/D(D is the number of variables)
	Distribution Indices	$\eta_{c}=20,\eta_{m}=20$

**Table 2:** IGD of Benchmark Functions.

Function	Indicators	MOPSO	MOPOA	POA-QPSO	MOQPSO	NSGA-II
ZDT1	AVE	0.00521	0.00526	0.00514	0.0224	0.1074
	Sd.	0.00052	0.00030	0.00017	0.001645	0.02677
ZDT2	AVE	0.61723	0.36845	0.09463	0.078	0.06613
	Sd.	0.24773	0.30148	0.20654	0.1775	0.03173
ZDT3	AVE	0.23244	0.23274	0.23267	0.2224	0.10609
	Sd.	0.00017	0.00016	0.00051	0.0009195	0.03557
ZDT4	AVE	2.920838	2.73731	0.275725	0.568448	1.68436
	Sd.	0.613359	2.61904	0.459765	0.402705	0.48221
ZDT6	AVE	0.00413	0.00341	0.00304	0.4767	0.12370
	Sd.	0.00131	0.00056	0.00035	0.2675	0.07283

**Table 3:** Spacing of Benchmark Functions.

Function	Indicators	MOPSO	MOPOA	POA-QPSO	MOQPSO	NSGA-II
ZDT1	AVE	0.00467	0.001380	0.001380	0.00111	0.00747
	Sd.	0.00066	0.00048	0.00049	0.00044	0.00061
ZDT2	AVE	0.00052	0.00293	0.00626	0.00626	0.00959
	Sd.	0.00161	0.00366	0.00829	0.00256	0.00366
ZDT3	AVE	0.00605	0.00961	0.00747	0.00711	0.00571
	Sd.	0.00107	0.00097	0.00076	0.00061	0.00054
ZDT4	AVE	0.16283	0.00004	0.04296	0.5828	0.00229
	Sd.	0.66562	0.00014	0.03529	0.6365	0.00020
ZDT6	AVE	0.01727	0.02835	0.12782	0.26623	0.15302
	Sd.	0.06667	0.10532	0.14955	0.23252	0.11199

**Table 4:** HV of Benchmark Functions.

Function	Indicators	MOPSO	MOPOA	POA-QPSO	MOQPSO	NSGA-II
ZDT1	AVE	0.723298	0.723229	0.723283	0.691749	0.58769
	Sd.	0.000045	0.000037	0.000029	0.002099	0.00415
ZDT2	AVE	0.424141	0.447062	0.447918	0.411216	0.35494
	Sd.	0.155946	0.000046	0.057452	0.000587	0.04224
ZDT3	AVE	0.600935	0.600836	0.601106	0.563216	0.57195
	Sd.	0.000027	0.00097	0.00169	0.003687	0.04050
ZDT4	AVE	0.000022	0.723383	0.549432	0.264043	0.00009
	Sd.	0.000121	0.00023	0.267999	0.246251	0.00027
ZDT6	AVE	0.390649	0.391049	0.391192	0	0.25104
	Sd.	0.000045	0.000012	0.000013	0	0.07899

**Table 5:** Walker configuration.

Parameter	Description
Orbital Altitude Range	Typically between 600 and 2000 km for Walker configuration, between 600 and 2000 km for polar configuration.
Inclination Range	Medium inclination, typically between 40° and 60° for Walker configuration, between 80° and 90° for polar configuration.
Number of Orbital Planes	Distributed among multiple satellites, ranging from dozens to hundreds.
Number of Satellites per Orbital Plane	the total count of satellites uniformly distributed along a single orbital plane within the constellation
Phase Factor Range	typically bounded by the number of orbital planes

**Table 6:** Experimental parameter range settings.

Layer	Main Function	Orbital Altitude Range (km)	Inclination Range (°)	Number of Orbital Planes	Number of Satellites per Orbital Plane	Phase Factor Range
Layer 1	Provides component mounting and wiring, primarily for signal routing and trace fanout routing capability.	400–800	50–70	10–20	15–20	0–(N−1)
Layer 2	Provides two-layer wiring, allows compensation for inadequate routing.	1000–1500	70–90	3–6	10–20	0–(N−1)

**Table 7:** Parameters of best LEO constellation.

Algorithm	Constellation	Orbit Altitude/km	Inclination/(°)	Coverage PDOP	PDOP	Number of Visible Satellites
POA-QPSO	W144/9/3	800	50	92.7 (global)	1.782	13.23
	W56/7/5	1426	82	93.5 (China)		
MOPSO	W144/9/1	400	47.2	82.1 (global)	2.042	11.05
	W77/7/3	1272	73	87.4 (China)		
NSGA-II	W154/14/2	448	50	91.1 (global)	2.241	11.48
	W72/12/4	1350	85	92.4 (China)		
MOPOA	W132/6/3	800	47	92.1 (global)	1.559	14.17
	W77/11/7	1345	84	93.2 (China)		

## Data Availability

The original contributions presented in this study are included in the article. Further inquiries can be directed to the corresponding author.
